# Internet-Delivered Psychoeducation (SCOPE) for Transition-Aged Autistic Youth: Pragmatic Randomized Controlled Trial

**DOI:** 10.2196/49305

**Published:** 2024-11-28

**Authors:** Anna Backman, Lise Roll-Pettersson, Are Mellblom, Elisabet Norman-Claesson, Emma Sundqvist, Eric Zander, Sarah Vigerland, Tatja Hirvikoski

**Affiliations:** 1 Socialstyrelsen National Board of Health and Welfare Stockholm Sweden; 2 Department of Special Education Stockholm University Stockholm Sweden; 3 Habilitation & Health Stockholm Healthcare Services Region Stockholm Stockholm Sweden; 4 Karolinska Institutet Center of Neurodevelopmental Disorders Department of Women's and Children's Health Karolinska Institutet Stockholm Sweden; 5 Centre for Psychiatry Research Karolinska Institutet Stockholm Sweden; 6 Child and Adolescent Psychiatry Stockholm Healthcare Services Region Stockholm Stockholm Sweden

**Keywords:** autism, internet based, young adult, intervention, digital communication, life satisfaction, codeveloped, ASD, autism spectrum disorder, autistic, RCT, randomized controlled trial, randomized, psychoeducation, patient education

## Abstract

**Background:**

Psychoeducation is a recommended first-line intervention for transition-aged autistic youth, but it has not been previously evaluated in an internet-delivered format. SCOPE (Spectrum Computerized Psychoeducation) is an 8-week individual, internet-delivered, therapist-supported psychoeducative intervention.

**Objective:**

This study aimed to investigate the effectiveness of SCOPE through a 3-armed randomized controlled trial. The intervention aims to increase participants’ understanding of autism and, in doing so, increase their quality of life (QoL).

**Methods:**

SCOPE was codeveloped with clinicians and autistic young adults. It contains 8 autism-related modules, each with (1) text describing the module topic, (2) four video vignettes with recurring characters who describe their lives and perspectives on the module topic, (3) a list of neurotypical characteristics related to the module’s topic, and (4) self-reflection using 3 or 4 questions about the module topic, answered by multiple-choice bullets and voluntary open-ended written comments. Participants were randomized (2:1:1) to SCOPE, an active control (web-based self-study), or treatment as usual (TAU). The primary outcome was participants’ autism knowledge, assessed using the Autism Spectrum Disorder Quiz, and secondary outcomes included acceptance of diagnosis, QoL, and symptoms of mental health problems. All outcomes were assessed at the baseline, postintervention, and 3-month follow-up time points, using mixed-effects models to assess change in outcome measures across time points.

**Results:**

Between 2014 and 2020, a total of 141 participants were randomized to 1 of the 3 treatment arms. The SCOPE participants had significantly greater autism knowledge gains at the posttreatment time point compared to TAU participants with a moderate effect size (*d*=0.47; *P*=.05); gains were maintained at the 3-month follow-up (*d=*0.46; *P*=.05). The self-study participants also had increased knowledge gains compared to TAU participants at the posttreatment time point with a moderate effect size (*d*=0.60; *P*=.03) but did not maintain these gains at the 3-month follow-up, and their autism knowledge scores returned to baseline (mean change score: –0.13, 95% CI –1.20 to 0.94; *P*=.81). In addition, SCOPE participants reported improved QoL at the postintervention (*d*=0.37, *P*=.02) and 3-month follow-up time points (*d*=0.60; *P*=.001), compared to the combined controls. The gained autism knowledge was not mirrored by changes in symptoms of anxiety or depression.

**Conclusions:**

Effective internet-delivered interventions may facilitate first-line service access to individuals who are unable or unwilling to use traditional health care interventions or who live in geographically remote locations. Additionally, an intervention such as SCOPE could impart and sustain the knowledge gained through psychoeducation in transition-aged autistic youth. For future research, qualitative studies could further our understanding of the lived experiences of intervention participation and outcomes after internet-delivered psychoeducation.

**Trial Registration:**

ClinicalTrials.gov NCT03665363; https://clinicaltrials.gov/study/NCT03665363

## Introduction

### Background

The transition to adulthood is challenging for many autistic individuals [1-4], with the transitioning period often coinciding with a loss of services, sometimes referred to as “dropping off a cliff,” rendering autistic individuals and their families without services [5,6]. To achieve the milestones of early adulthood as a transition-aged autistic youth necessitates skills for independence, which are reliant on self-knowledge about their own strengths and difficulties [1,7,8]. Psychoeducation is a well-established, evidence-based intervention for many psychiatric disorders, typically used to increase knowledge about their health condition, self-knowledge, and strategies in everyday life and to improve quality of life (QoL) [9,10]. Furthermore, psychoeducation aims to empower patients and actively engage them in their treatments as well as further service usage [10-12].

Indeed, psychoeducation is the recommended first-line intervention after a confirmed autism diagnosis [13-15]. However, the research field of psychoeducation for autistic individuals is still new and growing. In clinical settings, stepped-care models are recommended [14,16], by first providing a low-intensity intervention to most patients and later moving on to more intensive interventions if needed. In clinical practice in the United Kingdom [17] and in Sweden [13], the first step often entails studying psychoeducative materials, for example, reading an informative website or a pamphlet. The effects of self-study of informative websites about autism have not been evaluated; therefore, it is unknown whether it is an acceptable and effective intervention for autistic individuals. In addition, only a limited number of previous studies have described structured psychoeducational interventions for autistic individuals. In 2015, Gordon et al [18] conducted a randomized controlled trial (RCT) for autistic adolescents and their parents, with low attrition and high satisfaction among participants. Hidalgo et al [19] conducted an open trial for autistic adults and their close relations using group-based psychoeducational intervention, and Crane et al [20] used autistic-led peer groups; both were able to show low attrition as well as high satisfaction with program contents. However, in an open trial of a psychoeducational intervention for older autistic adults, Lenders et al [21] found a high level of attrition. Although all the abovementioned trials reported preliminary positive results of improved autism knowledge [18,19,21], QoL [19], and self-awareness [18], some studies had limitations such as small sample sizes [20,21] or no control group [19,21]. To further corroborate the evidence base of psychoeducation for autistic individuals, there is still a need for studies with rigorous trial designs, especially targeting older adolescents and emerging adults.

Psychoeducation using internet delivery could possibly contribute to a broader selection of intervention modalities and may also facilitate cognitive and communicative accessibility according to individual needs and preferences [22,23]. For example, autistic individuals explicitly ask for digital communication to improve comprehension, feel in control of the communication, and be able to express themselves freely and with humor [24,25]. Furthermore, recent studies of internet-delivered interventions aiming to promote health or behavioral changes for autistic adults showed positive preliminary results [26,27], and a continued use of technology-based interventions has been recommended in a review of technology-based interventions for autistic individuals [28]. Regarding internet-delivered psychoeducational interventions specifically, only 1 uncontrolled feasibility study has been published, showing good feasibility and acceptability for the internet-delivered psychoeducation SCOPE (Spectrum Computerized Psychoeducation) for transition-aged autistic youth [29].

In conjunction with examining the effectiveness of internet-delivered psychoeducation, it is necessary to consider the possible adverse effects of psychological interventions [30,31]. The tentative adverse effects of psychoeducation include lowered self-confidence and increased self-stigma, which are associated with reduced mental well-being [18,32]. Therefore, it is necessary to examine potentially harmful effects in terms of adverse changes in symptoms of anxiety, depression, and reduced self-acceptance following psychoeducation.

In summary, combining the asked-for, internet-based delivery with the recommended first-line psychoeducational intervention could potentially provide autism knowledge and empower transition-aged autistic youth. Therefore, promising interventions showing high acceptance and feasibility [29] need further evaluation in an RCT.

### Objectives

The objectives were to evaluate the effectiveness of SCOPE—a therapist-supported, internet-delivered psychoeducative program—using a 3-armed RCT design. We tested the hypotheses in Textbox 1.

**Hypotheses.**

- The effectiveness of internet-delivered psychoeducation (SCOPE [Spectrum Computerized Psychoeducation]) on autism knowledge (measured with an autism quiz) is superior to treatment-as-usual (TAU) or active web-based self-study comparators in a clinical outpatient setting.
- Acceptance of autism diagnosis and quality of life will increase at follow-up in SCOPE participants compared to TAU or active web-based self-study comparators.
- Symptoms of depression and anxiety will not increase following SCOPE participation.

## Methods

### Trial Design and Clinical Setting

This study is a 3-armed pragmatic RCT [33] of the internet-delivered psychoeducational intervention SCOPE for transition-aged autistic youth, conducted at Habilitation & Health (Stockholm Region outpatient disability services) in Stockholm, Sweden. The trial was registered at ClinicalTrials.gov (NCT03665363). Participants were enrolled between 2016 and 2021. Therapists were existing Habilitation and Health clinical staff trained in the SCOPE method.

### Ethical Considerations

In accordance with the ethical approval by the Swedish Ethical Review Authority (reference 2014/1868-31/5), prior to data collection, informed consent was obtained from all participants, and they were informed of their right to withdraw at any time. Participants were also informed about the purpose of the study and the methods used, and potential risks and benefits were discussed. Brief explanations about primary and potential secondary analyses of collected data were provided. Additionally, as approved by the Swedish Ethical Review Authority, all participants received a gift certificate of SEK 200 (US $22) after completing follow-up outcome measures.

### Participants’ Eligibility

Participants (n=141) were older adolescents and young adults, aged 16-25 years, with an autism diagnosis. Participants were recruited nationally through outpatient habilitation services, publicly funded psychiatric services, and private health care service providers. Swedish health care legislation allows nationwide service access, that is, individuals can seek health care in regions other than their listed address. All interested individuals were directed to a study recruitment website, provided by Habilitation & Health, to sign up and submit written consent. Professional staff and parents could assist participants in registering and accessing the study but could not apply on behalf of participants. Criteria for inclusion were (1) diagnosed autism (in accordance with the *Diagnostic Statistical Manual of Mental Disorders* or *International Classification of Diseases, Tenth Revision*) through community diagnosis with a documented IQ in the broad average range (ie, full-scale IQ>70); (2) aged 16-25 years; (3) sufficient Swedish language proficiency; and (4) the ability to log on and use the internet-based platform. Exclusion criteria were (1) diagnosed with acquired brain injury; (2) current problematic substance use; (3) severe suicidal ideation; (4) other severe psychiatric disorders (eg, psychosis); or (5) adverse personal circumstances (eg, being homeless) that could hinder participation. Ongoing pharmacological or other treatment was not considered a reason for exclusion.

### Procedure

#### Overview

An experienced licensed clinical psychologist working at Habilitation & Health (authors AB and ES) conducted the eligibility assessment using a video link. Community-based autism diagnoses were obtained through access to Region Stockholm medical records and signed diagnostic assessment reports. Autism diagnoses were validated using the Ohio State University Global Severity Scale for Autism (OARS-4) autism symptom checklist as an interview [34,35], and functional difficulties were validated using the World Health Organization Disability Assessment Schedule (version 2.0; WHODAS 2.0) interview [36]. In addition, self-rated autism traits (Social Responsiveness Scale, Second Edition [SRS-2]) [37] and symptoms of depression (Montgomery-Åsberg Depression Rating Scale—Revised [MADRS-R]) [38] were collected on the internet platform prior to the intake interview. SRS-2 was used to screen for the extent of autism traits (no cutoff was applicable), and MADRS-R was used to screen for depression symptoms (cutoff >35 was used as an exclusion criteria). The SCOPE intervention and active control condition of self-study consisted of weekly modules presented through the Swedish national online platform for telehealth services (*Stöd och Behandling* [SoB]) over 8 weeks. In contrast, treatment as usual (TAU) only involved the administration of data collection from the preintervention to follow-up time points. At follow-up, the active controls for self-study and TAU were asked if they wished to take part in SCOPE during the following term.

#### Randomization

Using the online tool randomizer.org, participants were randomized after baseline assessments individually to 1 of 3 conditions, with a 2:1:1 ratio to SCOPE, TAU, and active controls of self-study through 16 blocks of a minimum of 4 and a maximum of 13 participants. Baseline assessments were taken before the allocation of randomization was presented. Randomization sequences were conducted by author TH using a consecutively numbered list of the current block. The randomization results were given to author AB, who informed author AM of the allocation and in turn informed participants of their allocation via the SoB platform. Due to planning purposes and therapist availability, many participants had to wait until there were enough participants to be randomized to the next scheduled SCOPE block, ranging from a few days up to 70 days. Participants and therapists were blind to allocation at the preintervention assessments. When allocation was presented, no masking was achievable as therapists and participants were informed of the allocations and what they entailed.

#### Intervention

The SCOPE psychoeducative program was codeveloped with clinicians and autistic young adults, as described in more detail by Backman et al [29]. In addition, autistic designers created the visual presentation of some of the content. The contents have been carefully worded, containing information about autism and explicitly intending to empower the autistic participant. Following the feasibility study [29], SCOPE content was revised based on the participant feedback to amend language-related issues; the stringency of theme content; and, most saliently, include more information about autism-related strengths. SCOPE is an individual program that participants complete whenever and wherever they want. SCOPE has 8 autism-related themes (Introduction to Autism; Social Interaction; Behaviors & Interests; Theory of Mind; Central Coherence; Executive Functions; Intelligence & Memory; and Perception & Motor Skills). Each module focuses on one theme, containing (1) text describing the module topic; (2) four video vignettes with recurring transition-aged autistic characters who speak about their lives and perspectives on the module topic; (3) a bullet-point list of nonautistic characteristics related to the module's topic; and (4) self-reflection using 3 or 4 questions about the module topic, answered by multiple-choice bullets and voluntary open-ended written comments. In addition, the modules include evaluations of the content. Participants could adjust the pace at which they used the program contents but were asked to complete each module within a week.

The conception of the SCOPE treatment manual, therapist training, and supervision were conducted by the program developers (authors AM and ENC). The therapists were trained in the SCOPE manual, using a digital training program. They also received clinical supervision with their first 2 participants, and treatment fidelity was followed up. The manual included instructions for SCOPE’s technical aspects and provided the aims and perspectives relevant to SCOPE.

Therapists were health professionals, including clinical psychologists, special educators, social workers, and occupational therapists working in specialized outpatient habilitation service units for older autistic adolescents and autistic adults. SCOPE participants had weekly written contact with a therapist who supported them in interpreting the program content, providing brief motivational comments, dealing with practical issues that arose, and responding to participant comments using a phrasebook included in the SCOPE treatment manual for consistency in style and content of responses. In addition, the therapists reminded participants to log on by sending reminders and, on occasion, separate emails or SMS text messages if the system reminders went unopened.

The *self-study* condition entailed a standardized weekly message in SoB with links to 8 informative websites (1 link per week). The websites included professional, governmental, or stakeholder organization websites covering autism and related conditions (eg, the Swedish association for neurodevelopmental conditions Riksförbundet Attention or the Gillberg Neuropsychiatry Centre’s website). Self-study participants received initial information about allocation and what to expect from the self-study concept (ie, to open the weekly messages containing a link to an informative website) but no further personalized contact.

*TAU* entailed any ongoing support or intervention provided by health care services (eg, mental health or habilitation services). Participants reported on their ongoing health care contacts with a self-report form at baseline and 3-month follow-up (see Multimedia Appendix 1 for details). No restrictions regarding treatments were imposed.

#### Outcomes

All data were collected online using the Swedish national platform for internet-delivered interventions (SoB) at preintervention (baseline), postintervention, and 3-month follow-up time points. Data were then entered into an anonymized database.

#### Background and Demographic Data

Diagnostic assessment reports were extracted from the clinical patient records. Participants also completed a questionnaire covering sociodemographic and background information [29,39]. Finally, the participants' use of health care and other services was recorded using the background and demographic information questionnaire, collected at baseline and 3-month follow-up (Multimedia Appendix 1).

### Outcome Measures

#### Effectiveness

The primary outcome measure was autism knowledge. All participants completed the Autism Spectrum Disorder Quiz (ASD Quiz), which had shown acceptable reliability in a previous study [29], modified from a corresponding knowledge quiz [39,40]. The ASD Quiz consists of 16 questions related to facts about autism spectrum disorders; items are rated true, false, or unsure and subsequently assessed as either correct or incorrect, with correct answers summed for a total score out of 16. Higher scores indicate more autism knowledge. To evaluate the reliability of the ASD Quiz items, we applied the average item difficulty analysis, with 0.65 considered acceptable [41]. The average item difficulty at the preintervention time point was 0.62, while at the postintervention and follow-up time points, it was 0.69 and 0.69, respectively. We applied Kuder-Richardson 20 to estimate the internal consistency of the quiz; the coefficient α was 0.57 (n=141) at the preintervention time point, 0.63 (n=108) at the postintervention time point, and 0.59 (n=98) at follow-up.

Secondary effectiveness measures were self-rating questionnaires about the acceptance of diagnosis, QoL, as well as symptoms of mental ill-health. Acceptance of diagnosis was estimated using a modified version of the Acceptance and Action Questionnaire-II (AAQ-II) [29,42], rated from 0 to 7 on a Likert scale, with high scores indicating a low acceptance of the diagnosis. The modifications were done to enable the measurement of attitude (such as acceptance) toward one’s autism diagnosis through a questionnaire that had previously operationalized the measurement of “acceptance,” which in its original form meant to measure psychological flexibility. The modified items were as follows: (1) “My diagnosis makes it difficult for me to lead a life I could value”; (2) “I am afraid of my diagnosis”; (3) “I worry about not being able to control my worries and my feelings regarding my diagnosis”; (4) “My diagnosis prevents me from leading a fulfilling life”; (5) “My diagnosis creates problems in my life”; (6) “I feel uncomfortable with my diagnosis”; and (7) “My diagnosis gets in the way of my success.” Cronbach α at baseline for the acceptance of diagnosis was 0.85. Next, QoL was measured using The Brunnsviken Brief Quality of Life Scale (BBQ) [43], rated from 0 to 5 on a Likert scale for 2 aspects (satisfaction and importance) per QoL area, which was multiplied and then summed, with high scores indicating higher QoL. Cronbach α at baseline for the BBQ was 0.75. Finally, questionnaires for symptoms of mental health problems using the Swedish version of the Hospital Anxiety and Depression Scale (HADS) [44-46], rated from 0 to 3 on a Likert scale, with higher scores indicating more symptoms. Cronbach α at baseline for the HADS was 0.85.

#### Feasibility and Intervention Credibility

Adequate completion rate was defined a priori as >75% of participants completing SCOPE. Intervention completion was defined as having completed at least two-thirds of the modules (ie, 5/8, 62% of modules).

A modified version of the Treatment Credibility Scale (TCS) [47,48] was used to measure expectations of improvement and intervention credibility in SCOPE and self-study participants. The modified TCS is a visual analog scale rated from “Low credibility” or “Not at all” (0) to “High credibility” or “Very much” (10), with the mean of all items as the final score. The TCS was administered following the first module of SCOPE and self-study.

#### Adverse Events and Serious Adverse Events.

Adverse events (AEs) were defined in the individual Case Report Forms as “any inconvenience that participants spontaneously reported,” and serious AEs (SAEs) were defined as “anything that has required inpatient hospitalization.”

### Sample Size

A sample size of 126 participants was calculated to provide 80% power (1 – β) at a 2-sided 5% α level, with expectations of a moderate effect size. The effect size was estimated based on the results from the feasibility trial of SCOPE [29] and on a similar trial about psychoeducation for autistic adolescents [18]. To retain power with the expected attrition of 15%, we aimed to include 150 participants.

### Statistical Analyses

All statistical analyses were planned a priori; α was set at *P=*.05. The 2:1:1 ratio of randomization was chosen to enable a sufficient number of participants within the project time.

All variables were screened for violations of normality and sphericity; outcome data were approximately normally distributed. Statistical outliers for all outcome measures were screened using boxplots in SPSS (IBM Corp), and the few (n=7) extreme outliers (defined as outside the first or third quartile [SD 1.5]) were corrected by moving them to the closest whisker (ie, on the first or third quartile). Missingness was deemed completely at random for all outcome variables and time points using the Little missing completely at random test; we used allocation, age, gender, SRS-2, baseline BBQ, baseline HADS anxiety, and baseline HADS depression as assessment variables.

To determine changes between the intervention conditions (SCOPE vs TAU vs self-study) from the preintervention to postintervention time points and from the preintervention to follow-up time points, linear mixed models [49] used all available data without excluding cases with missing data. The models included random intercept, random time (slope), and other covariance structures; the model with the best fit was established using the log-likelihood ratio test. For outcomes where the slopes between self-study and TAU did not differ significantly (all outcomes apart from the ASD Quiz), the 2 control conditions were collapsed into one to increase power. For the ASD Quiz where the slopes of self-study and TAU differed significantly, post hoc tests were conducted to analyze the slopes of SCOPE with self-study and TAU, respectively. Between-group effect sizes, presented as Cohen *d*, were calculated by dividing the above-described coefficients of time×group interaction effect by the observed preintervention pooled SD [50]. The benchmark for Cohen *d* was based on the guidelines proposed by Cohen [51], where *d* values of 0.2, 0.5, and 0.8 are considered small, medium, and large effects, respectively.

### Community Involvement

The SCOPE intervention has been codeveloped and pilot-tested with autistic young adults. Several pilot iterations of the program were carried out online with the explicit purpose of codeveloping the program delivery (ie therapist involvement and feedback) and the contents evaluating the internet delivery, different aspects of the content (ie, module themes, phrasing, and video-vignette content), and therapist involvement and feedback. This work was carried out before the research projects commenced.

## Results

### Overview

**Figure 1:**
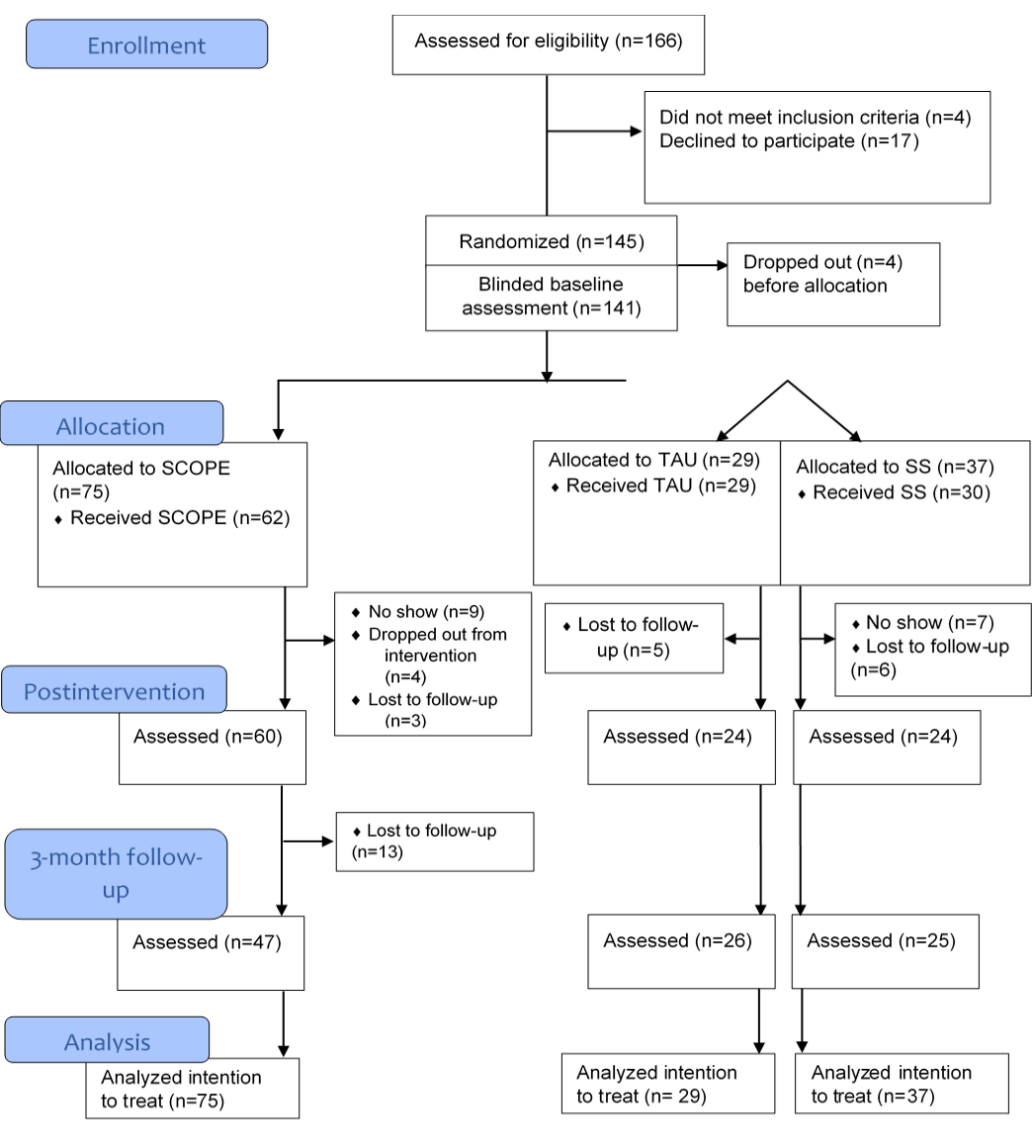
CONSORT (Consolidated Standards of Reporting Trials) flowchart of participants. SCOPE: Spectrum Computerized Psychoeducation; SS: self-study; TAU: treatment as usual.

A total of 166 participants were assessed for eligibility; see Figure 1 for the CONSORT (Consolidated Standards of Reporting Trials) flowchart. In total, 4 participants either did not meet inclusion or met exclusion criteria, and another 17 declined participation. The remaining (n=145) participants were enrolled in the study; they were asked to complete baseline measures and subsequently randomized. Baseline characteristics for SCOPE, self-study active controls, and TAU controls are presented in Table 1. Recruitment to the study spanned 5 years to achieve the final number of participants. The lengthy recruitment process was mainly due to slow rates of interested participants.

**Table 1:** Study participants’ sociodemographic and clinical characteristics at baseline, presented for the entire study group as well as by intervention condition.

Characteristics			All participants (N=141)		SCOPE^a^ (n=75)		Treatment as usual (n=29)		Self-study (n=37)
**Age (y), mean (SD)**			20.46 (2.96)		20.48 (3.07)		20.66 (3.10)		20.27 (2.67)
**Age at diagnosis (y), mean (SD)**			17.14 (4.38)		17.38 (3.93)		15.5 (4.76)		17.85 (4.82)
**WHODAS 2.0^b^ summary score, mean (SD)**			34.42 (11.23)		34.82 (11.15)		32.93 (12.09)		34.79 (10.9)
**SRS-2^c^ *t*-score, mean (SD)**			62.99 (7.67)		62.72 (7.68)		63.72 (7.43)		62.94 (7.86)
**OARS-4^d^ score, mean (SD)**			1.43 (0.39)		1.42 (0.37)		1.40 (0.43)		1.45 (0.41)
**Gender, female, n (%)**			92 (65.2)		54 (72)		18 (62.1)		20 (54.1)
**Psychiatric co-occurrence, n (%)**									
	At least 1 psychiatric condition^e^	89 (63.1)		53 (70.7)		13 (44.8)		23 (62.2)	
	At least 2 psychiatric conditions	35 (24.8)		20 (26.7)		7 (24.1)		8 (21.6)	
	ADHD^f^ diagnosis	48 (34.0)		28 (37.3)		7 (24.1)		13 (35.1)	
	Other NDDs^g,h^	15 (10.6)		9 (12)		1 (3.4)		5 (13.5)	
**Educational attainment^i^, n (%)**									
	Upper secondary	72 (54.1)		35 (46.7)		14 (50)		23 (62.2)	
	College or university	5 (3.6)		3 (4)		1 (3.6)		1 (2.7)	
**Living situation, n (%)**									
	Living independently	42 (29.8)		22 (29.3)		9 (31)		11 (29.7)	
	Living with partner	11 (7.8)		8 (10.7)		1 (3.4)		2 (5.4)	
	Living with parent	88 (62.4)		45 (60)		19 (65.5)		24 (64.9)	
**Occupation, n (%)**									
	Independent employment or student^j^	94 (66.7)		46 (62.3)		22 (75.9)		26 (70.3)	
	Supported employment	21 (14.9)		15 (20)		2 (6.9)		4 (10.8)	
	Not currently working or studying	8 (5.7)		5 (6.7)		2 (6.9)		1 (2.7)	

^a^SCOPE: Spectrum Computerized Psychoeducation.

^b^WHODAS 2.0: World Health Organization Disability Assessment Schedule (version 2.0).

^c^SRS-2: Social Responsiveness Scale, Second Edition.

^d^OARS-4: Ohio State University Global Severity Scale for Autism.

^e^At least 1 psychiatric condition entails *Diagnostic and Statistical Manual of Mental Disorders, Fifth Edition* psychiatric disorders other than the neurodevelopmental disorders.

^f^ADHD: attention-deficit/hyperactivity disorder.

^g^NDD: neurodevelopmental disorder.

^h^Other NDDs include communication disorders, specific learning disorders, tic disorders, and motor disorders.

^i^Educational attainment: ongoing educational attainment.

^j^Independent occupation or student: any work or education that is held without explicit support.

### Outcomes

Preintervention, postintervention, and follow-up assessments for all conditions based on the observed means and SDs are presented in Table 2, and interaction effects of between-group effects for preintervention to postintervention and preintervention to follow-up differences for all outcome measures are presented in Table 3.

**Table 2:** Observed means and SDs for SCOPE^a^, self-study active controls, and TAU^b^ at the preintervention, postintervention, and 3-month follow-up time points for primary (ASD Quiz^c^) and secondary outcomes (HADS^d^ anxiety, HADS depression, modified AAQ-II^e^, and BBQ^f^).

Outcome and treatment arm			Preintervention time point (baseline)			Postintervention time point			Follow-up	
		Participants, n		Score, mean (SD)	Participants, n		Score, mean (SD)	Participants, n		Score, mean (SD)
**ASD Quiz**										
	SCOPE	75		10 (2.7)	60		11.28 (2.17)	47		11.66 (2.59)
	Self-study	37		10.24 (1.85)	24		11.96 (1.46)	26		10.68 (2.04)
	TAU	29		9.62 (2.57)	24		10.13 (2.21)	25		10.27 (2.36)
**HADS anxiety**										
	SCOPE	75		10 (4.39)	60		9.33 (4.06)	47		9.17 (4.19)
	Self-study	37		9.70 (4.49)	24		10 (4.02)	26		9.52 (4.38)
	TAU	29		9.45 (5.06)	24		10.04 (4.33)	25		9.23 (4.02)
**HADS depression**										
	SCOPE	75		7.17 (4.16)	60		6.97 (4.45)	47		6.47 (4.03)
	Self-study	37		7.27 (3.89)	24		7.13 (4.09)	26		7.60 (3.45)
	TAU	29		6.00 (3.20)	24		6.13 (4.39)	25		5.62 (3.81)
**Acceptance of diagnosis (modified AAQ-II)**										
	SCOPE	75		24.83 (8.43)	60		22.63 (8.65)	47		21.38 (8.62)
	Self-study	37		22.81 (8.62)	24		22.21 (8.71)	26		22.60 (8.80)
	TAU	29		26.03 (8.32)	24		23.67 (8.40)	25		22.92 (8.26)
**BBQ**										
	SCOPE	75		41.77 (20.20)	60		45.51 (20.69)	47		49.30 (19.06)
	Self-study	37		44.84 (19.32)	24		45 (19.24)	26		43.32 (19.17)
	TAU	29		45.03 (17.50)	24		42.33 (17.40)	25		40.24 (18.32)

^a^SCOPE: Spectrum Computerized Psychoeducation.

^b^TAU: treatment as usual.

^c^ASD Quiz: Autism Spectrum Disorder Quiz.

^d^HADS: Hospital Anxiety and Depression Scale.

^e^AAQ-II: Acceptance and Action Questionnaire-II.

^f^BBQ: Brunnsviken Brief Quality of Life Scale.

**Table 3:** Between-group effect sizes (Cohen d) based on the estimates obtained in the hierarchical linear mixed models, presented together with the coefficient for mean change (least-squares means) and 95% CI for the interaction effect between time and intervention condition.

Measure	Comparison	Preintervention to postintervention difference					Preintervention to follow-up difference			
		Mean change scores	95% CI	Effect size^a^, *d*	95% CI	Mean change scores		95% CI	Effect size^a^, *d*	95% CI
ASD Quiz^b^	SCOPE^c^ vs TAU^d^	1.03	0.04 to 2.02	0.47	–0.01 to 0.96	1.15		0.15 to 2.15	0.46	–0.04 to 0.95
ASD Quiz	SCOPE vs self-study	–0.18	–1.18 to 0.83	–0.09	–0.57 to 0.39	1.27		0.25 to 2.30	0.53	0.02 to 1.03
HADS ANX^e^	SCOPE vs controls^f^	–0.95	–2.03 to 0.13	–0.23	–0.62 to 0.15	–.90		2.01 to 0.21	–0.22	–0.62 to 0.19
HADS DEP^g^	SCOPE vs controls	–.36	–1.57 to 0.86	–0.08	–0.47 to 0.30	–0.06		–1.82 to 0.68	–0.15	–0.55 to 0.25
Acceptance of diagnosis	SCOPE vs controls	–1.03	–3.04 to 0.98	0.12^h^	–0.26 to 0.50	–1.99		–4.07 to 0.09	0.23^h^	*-*0.15 to 0.61
BBQ^i^	SCOPE vs controls	7.21	1.79 to 12.62	0.37	–0.02 to 0.76	11.33		5.70 to 16.97	0.60	0.21 to 0.99

^a^Effect size measured by Cohen *d* (0.2 = small; 0.5 = moderate; 0.8 = large).

^b^ASD Quiz: Autism Spectrum Disorder Quiz.

^c^SCOPE: Spectrum Computerized Psychoeducation.

^d^TAU: treatment as usual.

^e^HADS ANX: Hospital Anxiety and Depression Scale—Anxiety subscale.

^f^Controls: self-study and TAU collapsed into one group for increased power.

^g^HADS DEP: Hospital Anxiety and Depression—Depression subscale.

^h^Sign of effect sizes have been changed so that positive scores indicate improvement.

^i^BBQ: Brunnsviken Brief Quality of Life Scale.

### Primary Outcome

**Figure 2:**
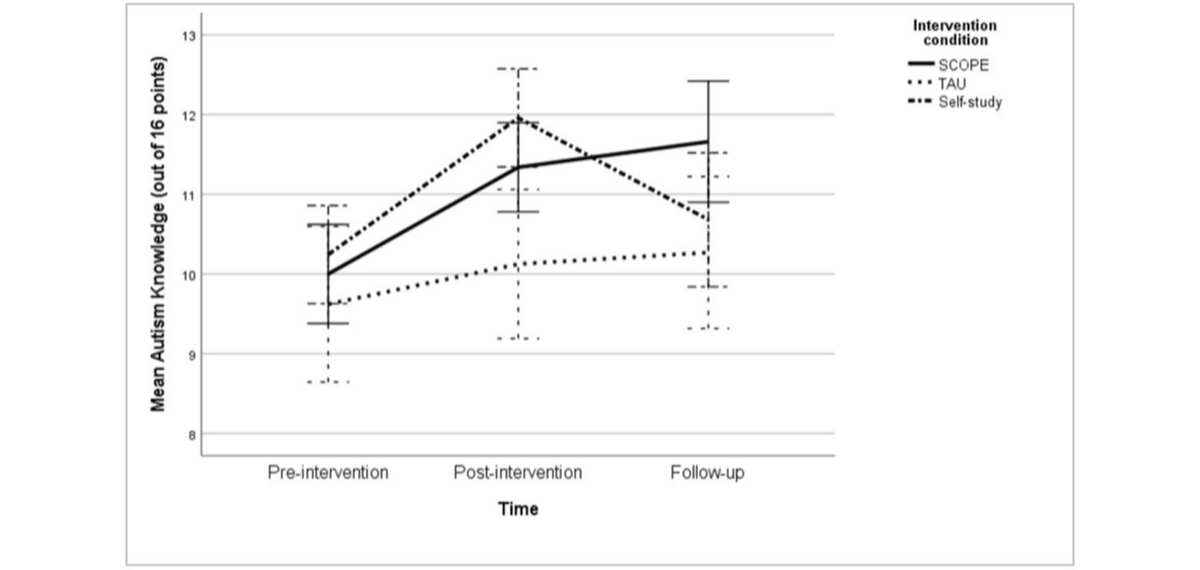
Multiple line graph of observed autism knowledge means at the preintervention, postintervention, and 3-month follow-up time points by intervention condition, with 95% CI error bars. SCOPE: Spectrum Computerized Psychoeducation; TAU: treatment as usual.

The primary outcome was autism knowledge, estimated using the ASD Quiz, which significantly increased for SCOPE and self-study participants from the baseline to postintervention time point compared to TAU, with a small effect size of *d*=0.47 (*P*=.04) between SCOPE and TAU at the postintervention time point. In addition, self-study participants had significantly higher autism knowledge compared to TAU at the postintervention time point, yielding a medium effect size of *d*=0.60 (mean change score: 1.20, 95% CI 0.11-2.30; *P*=.03). However, self-study participants' scores returned to baseline levels and did not differ significantly from TAU at follow-up (mean change score: –0.13, 95% CI –1.20 to 0.94; *P*=.81). In contrast, the ASD Quiz scores remained stable for SCOPE participants compared to self-study participants (*d*=0.53, *P*=.02) and TAU participants (*d*=0.48, *P*=.02) at 3-month follow-up. Figure 2 shows a visual representation of these interaction effects.

### Secondary Outcomes

The self-study active control and TAU conditions did not differ significantly for any secondary outcomes and were collapsed into one control group (see Table 3 for details).

The preintervention to postintervention increase in perceived QoL (the BBQ) was significantly larger in the SCOPE condition than in the combined controls. The between-group effect size was small at the postintervention time point (*d*=0.37, *P*=.02). However, QoL increased significantly at follow-up for SCOPE participants compared to combined controls, with a medium effect size of *d*=0.60 (*P*=.001).

Neither HADS Anxiety nor HADS Depression scores increased significantly between time points for SCOPE participants or the combined control condition. In addition, acceptance of diagnosis (modified AAQ-II) did not increase significantly between time points for SCOPE participants compared to the combined control condition.

### Feasibility and Intervention Credibility

Reported intervention credibility was similar for SCOPE participants (n=60; mean 7.69, SD (1.53) and self-study participants (n=30; mean 7.32, SD 1.90), measured after the first module.

At the postintervention time point, 2 (4%) of 62 SCOPE participants had not completed the intervention, whereas 6 (20%) out of 30 did not complete the self-study. In total, 5 (8%) out of 29 participants allocated to TAU dropped out of the study.

### AEs and SAEs

Although no SAEs were reported by SCOPE participants, there were 2 AEs spontaneously reported at the postintervention time point, where one participant dropped out of the study following module 6, referring to mental health problems, with no further contact after therapist attempts. However, it is unclear if the problems were related to SCOPE participation. The other participant completed SCOPE and all measurements but gave several spontaneous comments about feeling angry and commented that she or he felt some examples of autistic individuals were unfair and misrepresentative. The comments were given as part of the weekly written communication with the assigned therapist.

## Discussion

### Principal Findings

To the best of our knowledge, this is the first RCT to compare a structured, therapist-supported, internet-delivered psychoeducative intervention for transition-aged autistic youth against an active control condition (self-study) and TAU. In this trial, SCOPE was superior to self-study and TAU conditions at follow-up in providing autism knowledge and increasing QoL without increasing mental health problems. The findings of this RCT align with a previous open feasibility trial of SCOPE [29], in that the completion rate was high and the intervention was perceived as credible.

Our findings offer further support for the SCOPE intervention. In terms of our first hypothesis, we observed that SCOPE and self-study active control participants increased their autism knowledge, with small- to medium-sized effects, from the baseline to postintervention time points, whereas TAU did not. However, the increase in knowledge was only retained by SCOPE participants, showing a medium-sized effect at the 3-month follow-up, where self-study active controls participants’ knowledge returned to baseline rates. Furthermore, autism knowledge gains at follow-up were not related to preintervention treatment credibility, which was rated equally by the SCOPE and self-study groups. Instead, the maintained autism knowledge may be related to the carefully selected information, the codeveloper’s (ie, autistic emerging adults) influence on content and language, and the self-reflection questions possibly facilitating a more thorough cognitive processing of the learned information. Moreover, therapist guidance, through weekly written contact, is included in the SCOPE program but not in the self-study condition. However, this study cannot differentiate the active components in the interventions. Nevertheless, participants’ knowledge gains align with previous studies reporting similar results following group-based, structured psychoeducation for autistic adolescents and older adults [18,19]. In addition, a unique aspect of this study is the addition of an active comparator conducting self-studies, which has not previously been reported for similar interventions. Therefore, the results may have important clinical implications, as it is common for mental health care providers to give reading tips for self-study in the postdiagnostic phase. Based on the results of this study, self-study of internet-based reading materials without therapist support provided only temporary knowledge gains.

In addition to measuring autism knowledge, it is important to consider QoL as an important outcome following psychological interventions [31]. Improving QoL aligns well with the aims of psychoeducation—to empower and increase self-esteem. In salient theories on QoL, self-esteem is an integral part of QoL [52,53]. Moreover, Schalock [52] and the autism-focused review of QoL [54] see empowerment as a component of self-determination, which in turn is a component of QoL. As such, our second hypothesis about increased QoL following SCOPE was confirmed by the results, in that SCOPE was superior to controls in increasing QoL at the postintervention time point and even more so at follow-up. This was in line with the preliminary results of Hidalgo et al [19], showing a small increase in QoL in autistic adults following a structured psychoeducational group. Furthermore, enabling an increased sense of QoL is in line with up-to-date goals of treatment guidelines for autism [16] and calls to action in efficient postdiagnostic care [15], explicitly recommending psychoeducation as a first-line intervention after a confirmed autism diagnosis. Attempting to reframe and guide an individual’s perception of what autism is for them aligns with modern goals for psychoeducation [55]. A few studies on psychoeducation for autistic individuals have seen tentative effects on self-awareness and self-esteem [18,20], also described in the qualitative responses from the feasibility trial of SCOPE [29]. Increased knowledge about autism could act as a mediator of changes in self-perception and recognition of how autism impacts themselves and their environment. Although we were not able to conduct that kind of mediator analysis, this could be addressed in future studies.

Moreover, related to the third hypothesis, the increased knowledge among SCOPE and self-study participants was not coupled with increased symptoms of anxiety or depression. Although the primary goals of psychoeducation are not expected to have direct effects on specific symptoms such as anxiety and depression [9,55], and consequently, such effects have not previously been consistently found in the literature on psychoeducational interventions about autism [18,19,21,29,56]. Therefore, positive effects on symptoms of anxiety and depression were not expected to be improved in this study. Notably, the stability of anxiety and depression symptoms over time is an important finding, as psychoeducation may be associated with initial adverse psychological effects [17,32].

Furthermore, this study also partly supports the open feasibility trial of SCOPE [29], demonstrating high completion rates in the SCOPE. However, the recruitment of study participants was slow, with approximately 30 participants included every year (2016-2020). Given the apparent willingness of transition-aged autistic participants to receive interventions [6,57,58], we expected a higher flow of participants. The slow recruitment could suggest that we did not reach the intended target audience; perhaps, the intervention was perceived as too time-consuming or unappealing to transition-aged autistic youth, or the mode of study entry (application through the study website using e-identification) was cumbersome. Unfortunately, this study was unable to clearly identify the complex reasons for participant recruitment difficulties in this trial. A qualitative analysis of participants’ written responses and evaluations could deepen our understanding of the lived experiences of SCOPE participation and thus inform future implementation of similar programs in clinical practice.

### Limitations

This study has several limitations. First, the primary outcome measure (ASD Quiz) was based on previous similar measures [19,40,59], but it has not been psychometrically evaluated. However, no consensus regarding autism knowledge assessment exists, with considerable heterogeneity in the field [59], and the ASD Quiz used in this study has previously achieved acceptable reliability [29]. Although the knowledge gains were modest regarding absolute numbers, the effect size was medium at 3-month follow-up. However, a risk for ceiling effects in the primary outcome could have limited our ability to measure the treatment effects. Second, identifying secondary outcome measures available in Swedish for autistic individuals was challenging, especially at the inception of this study. For future studies, using outcome measures for self-esteem and empowerment would be valuable to evaluate the core aims of psychoeducation. Failing to measure these intervention outcomes misses the mark of understanding the potential effects of SCOPE within these domains. Third, detection bias may have been introduced due to assessors (the autistic individuals) being aware of intervention allocation at postintervention assessments [60]. Finally, the study’s generalizability may have been limited due to potential sampling issues. As noted in Table 1, the sample consists mainly of newly diagnosed female participants. The female majority may be reflective of findings maintaining that female individuals obtain their diagnosis later than male individuals [61,62], which may explain the oversampling of female participants in this type of intervention study. Moreover, autistic female individuals also report higher rates of mental health problems [63], perhaps contributing to more treatment-seeking behavior in general. Additionally, our participants reported many health care contacts (see Multimedia Appendix 1). Overall, our sample could represent a treatment-seeking *and* receiving subset of transition-age autistic youth that is not representative of those that have “fallen off a cliff” [6].

### Conclusions

This study shows that structured internet-delivered psychoeducation is effective in an outpatient context. The SCOPE program was superior to self-study active controls and TAU in imparting autism knowledge and increasing QoL. These results were maintained or further improved at the 3-month follow-up. In addition, providing internet-delivered services enables wide geographical accessibility and provides digital communication sought by transition-aged autistic youth.

## Appendix

Multimedia Appendix 1 Self-reported current interventions and psychotropic medications at baseline and 3-month follow-up, by treatment arm.

Multimedia Appendix 2 CONSORT-eHEALTH checklist (V 1.6.1).

## Abbreviations

AAQ-II: Acceptance and Action Questionnaire-II
AE: adverse event
ASD Quiz: Autism Spectrum Disorder Quiz
BBQ: Brunnsviken Brief Quality of Life Scale
CONSORT: Consolidated Standards of Reporting Trials
HADS: Hospital Anxiety and Depression Scale
MADRS-R: Montgomery-Åsberg Depression Rating Scale—Revised
OARS-4: Ohio State University Autism Rating Scale
QoL: quality of life
RCT: randomized controlled trial
SAE: serious adverse event
SCOPE: Spectrum Computerized Psychoeducation
SoB: Stöd och Behandling (Swedish national online platform for telehealth services)
SRS-2: Social Responsiveness Scale, Second Edition
TAU: treatment as usual
TCS: Treatment Credibility Scale
WHODAS 2.0: World Health Organization Disability Assessment Schedule (version 2.0)
