# Correction: Internet-Delivered Psychoeducation (SCOPE) for Transition-Aged Autistic Youth: Pragmatic Randomized Controlled Trial

**DOI:** 10.2196/72209

**Published:** 2025-02-10

**Authors:** Anna Backman, Lise Roll-Pettersson, Are Mellblom, Elisabet Norman-Claesson, Emma Sundqvist, Eric Zander, Sarah Vigerland, Tatja Hirvikoski

**Affiliations:** 1 Socialstyrelsen National Board of Health and Welfare Stockholm Sweden; 2 Department of Special Education Stockholm University Stockholm Sweden; 3 Habilitation & Health Stockholm Healthcare Services Region Stockholm Stockholm Sweden; 4 Karolinska Institutet Center of Neurodevelopmental Disorders Department of Women's and Children's Health Karolinska Institutet Stockholm Sweden; 5 Centre for Psychiatry Research Karolinska Institutet Stockholm Sweden; 6 Child and Adolescent Psychiatry Stockholm Healthcare Services Region Stockholm Stockholm Sweden

In “Internet-Delivered Psychoeducation (SCOPE) for Transition-Aged Autistic Youth: Pragmatic Randomized Controlled Trial” (J Med Internet Res 2024;26:e49305) the authors made two changes.

The affiliation of author Anna Backman has been changed from:

Child and Adolescent Psychiatry, Stockholm Healthcare Services, Region Stockholm, Stockholm, Sweden

to:

Socialstyrelsen, National Board of Health and Welfare, Stockholm, Sweden

In addition, the Corresponding Author’s address has been updated to:

Rålambsvägen 3, Stockholm, SE 106 30, Sweden

The correction will appear in the online version of the paper on the JMIR Publications website on February 10, 2025, together with the publication of this correction notice. Because this was made after submission to PubMed, PubMed Central, and other full-text repositories, the corrected article has also been resubmitted to those repositories.

