# Supplementary material for: Internet-Delivered Psychoeducation (SCOPE) for Transition-Aged Autistic Youth: Pragmatic Randomized Controlled Trial
Source: J Med Internet Res. 2024 Nov 28;26:e49305. doi: 10.2196/49305 (PMC11638691; doi:10.2196/49305)
Supplement: Multimedia Appendix 1 [file jmir_v26i1e49305_app1.docx]

|  | All participants | | SCOPE | | Treatment as Usual | | Self-study | |
| --- | --- | --- | --- | --- | --- | --- | --- | --- |
|  | baseline  n = 141 | follow-up  n = 98 | baseline  n = 75 | follow-up  n = 47 | baseline  n = 29 | follow-up  n = 25 | baseline  n = 37 | follow-up  n = 26 |
|  | n (%) | n (%) | n (%) | n (%) | n (%) | n (%) | n (%) | n (%) |
| **Interventions** |  |  |  |  |  |  |  |  |
| Regular MD visits | 36 (25.5) | 22 (22.4) | 18 (24) | 15 (31.9) | 11 (37.9) | 6 (24) | 7 (18.9) | 1 (3.8) |
| Psychotherapy | 50 (35.4) | 19 (19.4) | 28 (37.3) | 10 (21.3) | 13 (44.8) | 6 (24) | 9 (24.3) | 3 (11.5) |
| Group therapy | 4 (2.8) | 4 (4.1) | 3 (4) | 2 (4.3) | 0 | 1 (4) | 1 (2.7) | 1 (3.8) |
| Municipal support | 16 (11.3) | 8 (8.2) | 8 (10.7) | 7 (14.9) | 3 (10.3) | 1 (4) | 5 (13.5) | 0 |
| Social Services | 6 (4.3) | 3 (3.1) | 2 (2.7) | 1 (2.1) | 2 (6.9) | 0 | 2 (5.4) | 2 ( 7.7) |
| Cognitive aid | 15 (10.6) | 11 (11.2) | 9 (12) | 7 (14.9) | 4 (13.8) | 1 (4) | 2 (5.4) | 3 (11.5) |
| **Psychotropic medications** |  |  |  |  |  |  |  |  |
| Stimulants | 23 (16.3) | 18 (18.4) | 13 (17.3) | 9 (19.1) | 6 (20.7) | 5 (20) | 4 (10.8) | 4 (15.4) |
| SSRIs | 57 (40.4) | 35 (35.7) | 30 (40) | 22 (46.8) | 15 (51.7) | 7 (28) | 12 (32.4) | 6 (23) |
| Attarax | 16 (11.3) | 15 (15.3) | 8 (10.7) | 9 (19.1) | 5 (17.2) | 3 (12) | 3 (8.1) | 3 (11.5) |
| Benzodiazepines | 7 (5) | 3 (3.1) | 4 (5.3) | 2 (4.3) | 0 | 0 | 3 (8.1) | 1 (3.8) |
| Sleep | 30 (21.3) | 16 (16.3) | 12 (16) | 8 (17) | 12 (41.3) | 5 (20) | 6 (16.2) | 3 (11.5) |
| Other | 49 (34.8) | 30 (30.6) | 33 (44) | 15 (31.9) | 11 (37.9) | 7 (28) | 5 (13.5) | 8 (30.8) |
